# Association between voice handicap and life satisfaction in older adults following dental implant treatment

**DOI:** 10.3389/fpsyg.2026.1787234

**Published:** 2026-03-26

**Authors:** Agit Simsek, Selman Bolukbasi, Irfan Ustundag, Recep Akmese, Yunus Cetiner

**Affiliations:** 1 Department of Speech and Language Therapy, Faculty of Health Sciences, Inonu University, Malatya, Türkiye; 2 Department of Gerontology, Faculty of Health Sciences, Inonu University, Malatya, Türkiye; 3 Department of Oral and Maxillofacial Surgery, Faculty of Dentistry, Inonu University, Malatya, Türkiye

**Keywords:** aged, dental implants, perception, personal satisfaction, phonetics

## Abstract

**Background:**

Communication-related functional outcomes in later life remain insufficiently addressed despite their relevance to adaptive capacity and psychosocial well-being. This study examined the association between perceived voice-related functional limitations and life satisfaction among older adults following dental implant treatment, with attention to indicators of age-related vulnerability.

**Methods:**

This cross-sectional study included adults aged 60 + years who had received dental implant treatment. Perceived voice-related functional limitations were assessed using the Voice Handicap Index-10, while life satisfaction was measured with the Satisfaction with Life Scale. Multivariate analyses examined independent associations while controlling for sociodemographic and health-related variables.

**Results:**

In 285 older adults (mean age 67.7 ± 6.1 years), bivariate analysis revealed a significant negative association between perceived voice-related functional limitation and life satisfaction (Spearman’s rho = −0.24, *p* < 0.001). In multivariable analysis, psychotropic medication use (OR = 3.19, 95% CI: 1.63–6.23, *p* = 0.001) and lower educational attainment were independently associated with perceived voice handicap, suggesting increased vulnerability in specific subgroups.

**Conclusion:**

Perceived voice-related functional limitations represent an important correlate of life satisfaction in later life, with the relationship likely influenced by broader vulnerability indicators. These findings underscore the importance of interdisciplinary approaches integrating dental care, speech therapy, and gerontological perspectives to support adaptive capacity in older adults.

## Graphical abstract

**Figure:**
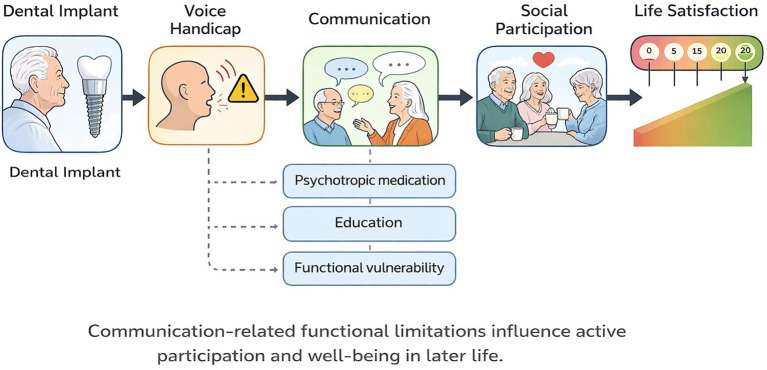

## Introduction

The rapid increase in the older population worldwide has led to a growing emphasis on holistic health approaches aimed at improving quality of life in this age group (Petersen and Yamamoto, 2005). During the aging process, oral and dental health is not limited solely to nutrition and masticatory functions; rather, it is regarded as a multidimensional health indicator that directly affects an individual’s social, psychological, and phonational (voice production) capacities. Dental implant treatments, which are widely preferred in the rehabilitation of tooth loss, provide not only esthetic and functional improvements in older adults but also positively influence self-confidence, social communication capacity, and overall quality of life (Takeuchi et al., 2023; Kalyoncuoğlu and Ergüven, 2023). The literature emphasizes that implant supported prostheses play a much more critical role than conventional prostheses in terms of psychosocial rehabilitation and speech quality (Takeuchi et al., 2023; Heydecke et al., 2004).

Voice, as one of the fundamental components of communication, is directly related to an individual’s manner of self-expression and participation in social life. However, the reshaping of the speech organs following dental implant procedures, particularly the lips, tongue, palate, and dental alignment, may lead to a subjective perception of a voice related “handicap” in some individuals (Lundqvist et al., 1992). Such changes in phonational processes may restrict speaking behavior, result in the avoidance of communication, foster a negative self-perception of voice, and ultimately lead to social isolation (Rouxel et al., 2016). This highlights the need for dental implant applications to be evaluated not only in terms of physiological outcomes but also with respect to their psychosocial consequences using both objective and subjective criteria.

Life satisfaction in older adults is shaped as a holistic outcome of biological, psychological, and social factors (Durak et al., 2010). Perceived limitations related to one’s own voice, communication difficulties, and the resulting social withdrawal may be among the important variables that negatively affect life satisfaction. Although the effects of implant treatments on esthetics and masticatory function have been extensively examined in the dental literature, studies investigating the relationship between perceived post treatment voice handicap and life satisfaction remain quite limited (Strassburger et al., 2004).

In this context, the present study aims to investigate the relation of perceived voice handicap with life satisfaction in individuals aged 60 years and older who have undergone dental implant treatment at the Department of Oral, Dental, and Maxillofacial Surgery, Faculty of Dentistry, Inonu University. Within the scope of the study, participants’ scores on the Voice Handicap Index 10 (VHI-10) and the Satisfaction with Life Scale (SWLS) will be evaluated, and the effects of variables such as gender, age, educational level, and presence of systemic diseases on this relationship will be analyzed.

While existing literature has examined the impact of dental implant treatment on masticatory function, esthetics, and general quality of life, the specific relationship between perceived voice-related functional limitations and life satisfaction in this population remains understudied. Moreover, little is known about which subgroups of older implant recipients may be most vulnerable to communication-related challenges following treatment. This study addresses these gaps by: (1) examining the association between perceived voice handicap and life satisfaction in a sample of older adults post-implant treatment, and (2) identifying sociodemographic and clinical factors associated with increased voice-related vulnerability.

The findings of this study are expected to raise awareness of the psychosocial dimensions of dental implant treatments and to contribute to filling the gap in the literature. Accordingly, the main hypothesis of the study is that as the level of perceived voice handicap increases in older individuals receiving dental implant treatment, their overall level of life satisfaction decreases.

## Methods

### Study design

This is a cross-sectional, observational, analytical study that was conducted to investigate the influence of voice handicaps on life satisfaction in older individuals following dental implant treatment. The research was designed in the framework of a correlational survey model, and the relations between dependent and independent variables were tested using multivariate statistical methods.

### Study population and sample

The population of the study consisted of individuals aged 60 years and older who had undergone dental implant procedures within the last 6 years at the Department of Oral, Dental, and Maxillofacial Surgery, Faculty of Dentistry, Inonu University. The criteria for inclusion in the study were being 60 years of age or older, having a history of at least one dental implant treatment, and volunteering to participate in the study. Those participants who left the questionnaires incomplete or preferred to withdraw from this study were excluded. A sample size of 285 individuals that matched all the inclusion criteria agreed to participate in this study. The required sample size was deemed sufficient according to the number of observations per variable recommended for logistic and linear regression analyses. The sample was determined using a purposive sampling method, as it was not possible to reach the entire population.

### Data collection instruments

#### Demographic information form

This questionnaire was prepared with 22 structured questions about the participants’ age, gender, marital status, educational level, family type, number of children, place of residence, number of people in the household, type of house, living arrangement, time spent with family and friends, number of implants, duration of implant treatment, use of prostheses prior to implantation, opinions about dental implants, interest in oral and dental health, ability to speak in public, information on chronic diseases, use of psychotropic medication, perceived health status, and smoking and economic status. In the evaluation of the economic status, the current minimum wage was considered.

#### Voice handicap index

VHI-10 was used to assess participants’ voice related functional, physical, and emotional limitations. This scale is the short form of the original 30 item Voice Handicap Index developed by Jacobson et al. (1997) and was introduced into clinical use following validity and reliability studies conducted by Rosen et al. The VHI-10 is a unidimensional measurement tool that quantitatively evaluates the subjective impact of voice disorders on individuals’ daily lives (Rosen et al., 2004). The total VHI-10 score is obtained from 10 items, each scored between 0 and 4, yielding a total score range of 0–40. Higher scores indicate a greater level of perceived voice handicap (Kılıç et al., 2008). In the current sample, the VHI-10 demonstrated good internal consistency (Cronbach’s α = 0.89).

In this study, the scores obtained from the scale were used according to two different analytical approaches. First, the severity of voice handicap was treated as a continuous variable to evaluate its continuous distribution. Second, in order to enhance clinical relevance and enable multivariable modeling aimed at identifying risk factors, the scores were converted into a binary categorical variable based on the cutoff value recommended in the literature. Accordingly, individuals with a total VHI-10 score of ≥11 were classified as having a voice handicap.

This dichotomous variable was used as the dependent variable in multivariable logistic regression analyses conducted to identify sociodemographic and clinical factors affecting the presence of voice handicap. Continuous VHI-10 scores were included in multivariable linear regression analyses to evaluate factors influencing life satisfaction.

#### Satisfaction with life scale

SWLS was used to assess life satisfaction, which represents the cognitive component of participants’ subjective well-being. The scale was developed by Diener et al. and is a unidimensional measurement tool based on individuals’ global and holistic evaluations of their lives (Diener et al., 1985). The validity and reliability of the Turkish adaptation of the SWLS have been previously established, and it is considered methodologically appropriate for use in the older population (Dağlı and Baysal, 2016). The SWLS consists of 5 items, each rated on a 1–5 Likert scale (from strongly disagree to strongly agree). The total score obtainable from the scale ranges from 5 to 25, with higher scores indicating greater life satisfaction. Internal consistency in the present sample was satisfactory (Cronbach’s α = 0.85). In this study, the total SWLS score was treated as a continuous variable after examining its distributional properties.

To identify factors influencing life satisfaction, the total SWLS score was included as the dependent variable in multivariable linear regression analyses. Voice handicap level (continuous VHI-10 score), sociodemographic variables, and clinical characteristics were entered into the model as independent variables. The assumption of multicollinearity was assessed using variance inflation factor (VIF) values, and model fit was reported using the coefficient of determination (R^2^) and adjusted R^2^. Statistical significance was set at *p* < 0.05.

### Data collection process

Data were collected using a face-to-face interview method. During the interviews, the questions included in the study were presented to the participants in a predetermined order and without the use of any leading expressions. The responses obtained were transferred by the researchers to a Google Forms–based digital data recording system after the interviews. Mandatory fields were defined in the digital form, and incomplete data entry was not permitted. Participants were verbally informed about the purpose and scope of the study, and written informed consent was obtained from all participants. Throughout the data collection process, strict adherence to the principles of anonymity and confidentiality was ensured. Participant information was stored in coded form, and no identifying data were recorded.

### Ethical considerations

Ethical approval for the study was obtained from the Inonu University Health Sciences Scientific Research Ethics Committee at its session dated 16 September 2025, with decision number 2025/8217. The study was conducted in full accordance with the principles of the Declaration of Helsinki throughout the research process.

### Statistical analysis

Statistical analyses were performed using IBM SPSS Statistics version 26. The distributional properties of continuous variables were assessed using the Kolmogorov–Smirnov test, along with skewness and kurtosis coefficients, and the variables were found not to follow a normal distribution. Accordingly, all continuous variables were analyzed using non-parametric methods. Continuous variables were described as median (minimum–maximum), while categorical variables were presented as numbers and percentages. For comparisons between two groups, the Mann–Whitney U test was used for continuous variables, and for comparisons among more than two groups, the Kruskal–Wallis test was applied. Associations between categorical variables were evaluated using the chi-square test or Fisher’s exact test, as appropriate.

To identify factors associated with the presence of voice handicap, a dichotomous variable (present/absent) created based on the VHI-10 cutoff value was used as the dependent variable, and multivariable logistic regression analysis was performed. Independent variables included in the model were selected from those showing significance at the *p* < 0.20 level in univariable analyses. Regression results were reported as odds ratios (ORs), 95% confidence intervals (CIs), and *p* values. Model fit was assessed using the Hosmer–Lemeshow goodness-of-fit test.

To evaluate factors influencing life satisfaction, the total score of the SWLS was treated as the dependent variable, and multivariable linear regression analysis was conducted. Due to violations of the normality assumption, robust standard errors were used in the regression analyses. Multicollinearity was assessed using VIF values. The explanatory power of the model was evaluated using *R*^2^ and adjusted *R*^2^ values. In all statistical analyses, a two-tailed *p* value of < 0.05 was considered statistically significant.

## Results

The normality of the VHI-10 and SWLS scores was assessed using the Kolmogorov–Smirnov and Shapiro–Wilk tests. The assumption of normal distribution was not met for either scale (*p* < 0.001). Therefore, non-parametric tests were used in the analyses.

The study was conducted with 285 older adults who had received dental implant treatment. The mean age of the participants was 67.69 ± 6.09 years, with the youngest participant aged 60 years and the oldest 97 years. Of the sample, 54.7% (*n* = 156) were male and 45.3% (*n* = 129) were female. The majority of participants were married (76.8%) and lived in a nuclear family structure (65.3%). Regarding educational level, 19.6% of the participants were illiterate, 9.1% were literate without formal education, 24.2% had completed primary school, 18.2% middle school, 18.2% high school, and 10.5% were university graduates. Most participants lived in apartment buildings (49.1%) and in urban areas (65.3%). The mean SWLS score was 13.9 ± 4.7. In terms of VHI-10, the presence of voice handicap was identified in 34.7% of the participants.

No significant difference was found in the presence of VHI-10 according to gender (χ^2^ = 0.02, *p* = 0.50). Similarly, no statistically significant associations were observed between VHI and marital status or family type (*p* > 0.05). A significant difference was found between educational level and VHI-10 (χ^2^ = 50.5, *p* = 0.01). The prevalence of voice handicap was markedly higher among illiterate individuals (73.2%) compared with other educational groups, while it was relatively lower among university graduates, suggesting that educational disadvantage may compound vulnerability to perceived voice problems. Housing type and place of residence showed significant differences with respect to VHI-10. The prevalence of voice handicap was 43.4% among individuals living in detached houses, compared with 25.7% among those living in apartments (*p* = 0.001). Participants living in rural areas had a higher prevalence of VHI-10 (47.5%) than those residing in urban areas (28.0%) (*p* = 0.001). As time spent with friends or family members increased, the prevalence of voice handicap decreased. The rate of VHI-10 was 41.3% among individuals with 0–2 h of daily social interaction, whereas it declined to 24.2% among those with 5 h or more of interaction per day (*p* = 0.03), indicating that greater social engagement may serve a protective role, possibly through increased communicative practice and reinforcement of speech confidence. The prevalence of voice handicap was 62.9% among individuals using psychiatric medications, which was significantly higher compared with those not using such medications (*p* < 0.001); this strong association may reflect both medication side effects on oral-motor function and the general vulnerability of individuals with mental health conditions. Similarly, the rate of VHI was 40.0% in individuals with chronic diseases, compared with 25.0% in those without chronic conditions (*p* = 0.007). Among smokers, the prevalence of voice handicap was 41.7%, whereas it was 28.1% among non-smokers (*p* = 0.01). Individuals who did not pay adequate attention to oral and dental health had a significantly higher prevalence of VHI-10 (*p* = 0.009). The mean number of implants was 4.42 ± 3.12. No significant association was found between the number of implants and VHI-10 or SWLS scores (*p* > 0.05). The mean duration of implant treatment was calculated as 2.73 ± 1.95 years. A statistically significant association was observed between treatment duration and life satisfaction scores, with SWLS scores increasing as treatment duration increased (*p* = 0.02). However, no significant relationship was identified between implant treatment duration and voice handicap. The prevalence of voice handicap was significantly higher among individuals who had used prostheses prior to implant treatment (39.9%) compared with those who had not (29.2%) (*p* = 0.04). Among participants expressing negative opinions about implant treatment, the prevalence of voice handicap was 56.9%, whereas it was 29.9% among those with positive opinions (*p* < 0.001).

Life satisfaction scores did not differ significantly according to gender, marital status, educational level, or family type (*p* > 0.05). However, SWLS scores were significantly lower among individuals who smoked (12.5 ± 4.5) compared with non-smokers (15.2 ± 4.6) (*p* < 0.001). Participants with a good perception of their health had significantly higher life satisfaction scores (14.3 ± 5.1) than those who perceived their health as moderate or poor

(13.5 ± 4.4) (*p* < 0.001). A weak, negative, and statistically significant correlation was found between the Voice Handicap Index and the Satisfaction with Life Scale (Spearman’s rho = −0.24, *p* < 0.001), indicating that life satisfaction decreased as the level of voice handicap increased (Table 1).

**Table 1:** Comparison of participants’ sociodemographic characteristics according to VHI-10 and SWLS scores.

Sociodemographic status				VHI	*P*	SWLS	*P*
Features		*N*	%				
Total		285	100	99 (34.7%)		13.9 ± 4.7	
Gender	Male	156	54.7	54 (34.6%)	*X* = 0.02* p = 0.5	13.4 ± 4.8 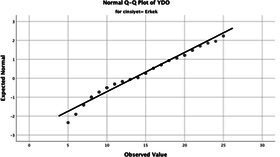	*Z* = −1.8** p = 0.7
	Female	129	45.3	45 (34.9%)		14.5 ± 4.6 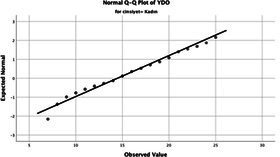	
Age	Mean ± SD	67.69 ± 6.09 (60–97)		68.36 ± 6.45	*Z* = −1.5** p = 0.1		S.rho = −0.15**** p = 0.01
	Median/mode	67/60					
Education level	Illiterate	56	19.6	41 (73.2%)	*X* = 50.5* p = 0.01	14.8 ± 4.5 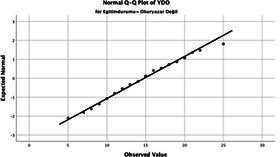	*H* = 9.3*** p = 0.09
	Literate	26	9.1	10 (38.5%)		13.5 ± 4.7 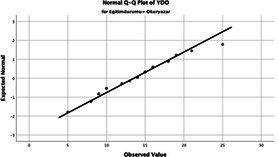	
	Primary	69	24.2	14 (20.3%)		14.4 ± 3.3 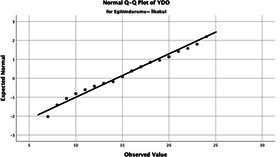	
	Secondary	52	18.2	11 (21.2%)		13.0 ± 4.9 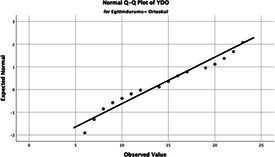	
	High school	52	18.2	12 (23.1%)		12.7 ± 4.7 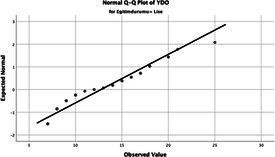	
	University	30	10.5	11 (36.7%)		14.9 ± 5.7 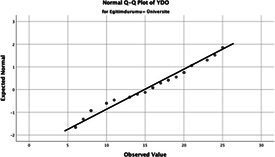	
Marital status	Single	66	23.2	17 (40.9%)	*X* = 1.4* p = 0.14	13.8 ± 4.9 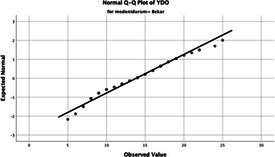	*Z* = −0.2** p = 0.9
	Married	219	76.8	72 (32.9%)		13.9 ± 4.7 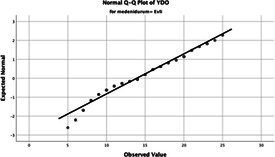	
Family type	Nuclear	186	65.3	59 (31.7%)	*X* = 2.1* p = 0.9	14.2 ± 4.7 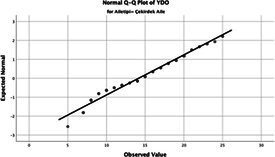	*Z* = −1.5** p = 0.2
	Extended	99	34.7	40 (40.4%)		13.4 ± 4.8 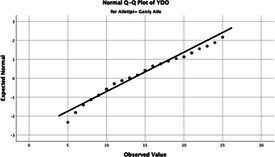	
Number of children alive	Mean ± SD	3.09 ± 2.39 (0–18)		3 ± 3.19	*Z* = −2.2** p = 0.02		S.rho = −0.03**** p = 0.5
	Median/mode	3/3					
How many people live in the house	Mean ± SD	3.29 ± 2.3 (1–23)		3.63 ± 2.6	*Z* = −1.1** p = 0.3		S.rho = −0.08**** p = 0.2
	Median/mode	3/2					
House type	Apartment	140	49.1	36 (25.7%)	*X* = 9.9* p = 0.001	14.2 ± 4.9 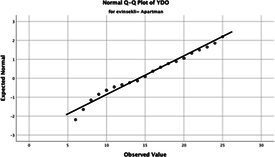	*Z* = −1.04** p = 0.3
	Detached	145	50.9	63 (43.4%)		13.6 ± 4.6 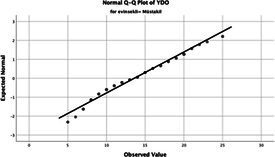	
Lifestyle	Urban	186	65.3	52 (28.0%)	*X* = 10.9* p = 0.001	13.7 ± 4.9 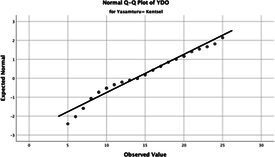	*Z* = −0.8** p = 0.4
	Rural	99	34.7	47 (47.5%)		14.2 ± 4.4 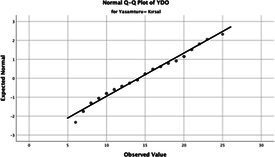	
Time with friends or family	0–2	92	32.2	38 (41.3%)	*X* = 4.98* p = 0.03	14.1 ± 4.8 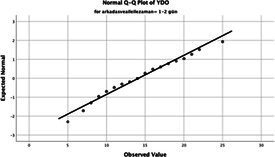	fv*H* = 1.9*** p = 0.4
	3–4	127	44.6	45 (35.4%)		13.4 ± 4.4 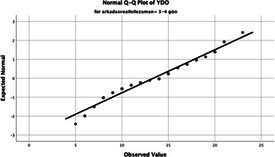	
	5+	66	23.2	16 (24.2%)	14.5 ± 5.2 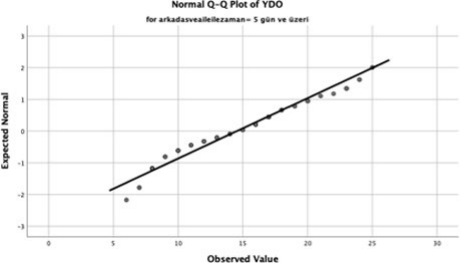
Number of implants	Mean ± SD	4.42 ± 3.12 (1–16)		4.12 ± 3.14	*Z* = −1.7** p = 0.09		S.rho = −0.03**** p = 0.6
	Median/mode	3/2					
Implant treatment (year)	Mean ± SD	2.73 ± 1.95 (1–12)		3.23 ± 2.09	*Z* = −3.2** p = 0.02		S.rho = −0.03**** p = 0.6
	Median/mode	2/1					
Prosthetic use before implant	Yes	148	51.9	59 (39.9%)	*X* = 3.57* p = 0.04	13.9 ± 4.5 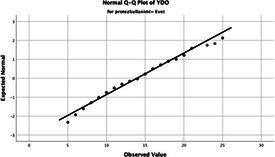	*Z* = −0.25** p = 0.8
	No	137	48.1	40 (29.2%)		13.8 ± 4.9 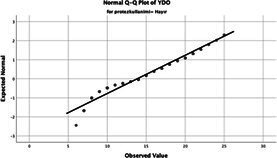	
Opinion about implant	Positive	234	82.1	70 (29.9%)	*X* = 13.4* p < 0.001	13.8 ± 4.9 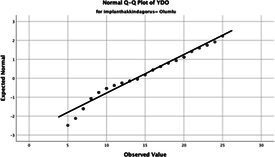	*Z* = −0.54** p = 0.6
	Negative	51	17.9	29 (56.9%)		14.1 ± 4.0 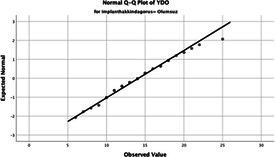	
Pay attention to oral and dental health	Yes	238	83.5	75 (31.5%)	*X* = 6.6* p = 0.009	14.0 ± 4.8 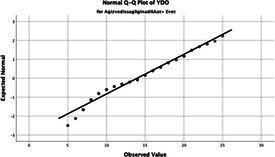	*Z* = −0.7** p = 0.5
	No	47	16.5	24 (51.1%)		13.5 ± 4.7 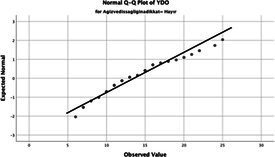	
Public speaking	Yes	229	80.4	72 (31.4%)	*X* = 5.6* p = 0.02	13.8 ± 4.8 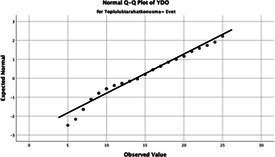	*Z* = −0.67** p = 0.5
	No	56	19.6	27 (48.2%)		14.2 ± 4.5 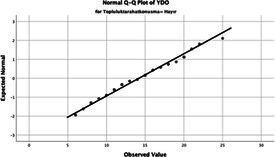	
Psychiatric medication	Yes	62	21.8	39 (62.9%)	*X* = 27.7* p < 0.001	13.0 ± 4.1 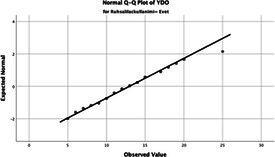	*Z* = −1.64** p = 0.09
	No	223	78.2	60 (26.9%)		14.2 ± 5.0 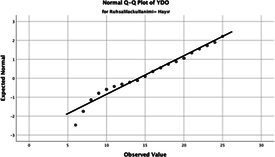	
Chronic disease	No	185	64.9	25 (25.0%)	*X* = 6.4* p = 0.007	13.6 ± 4.9 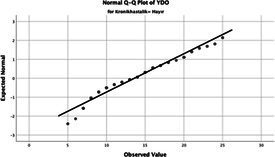	*Z* = −1.76** p = 0.08
	Yes	100	35.1	74 (40.0%)		14.4 ± 4.3 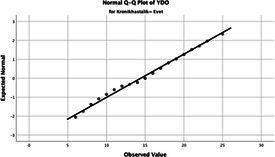	
	Mean ± SD	1.65 ± 0.47 (1–2)		2.05 ± 1.09	*Z* = −0.2** p = 0.8		S.rho = −0.1**** p = 0.07
	Median/mode	2/2					
Cigarette	Yes	139	48.8	58 (41.7%)	*X* = 5.8** p = 0.01	12.5 ± 4.5 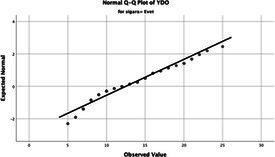	*Z* = −4.9** p < 0.001
	No	145	51.2	41 (28.1%)		15.2 ± 4.6 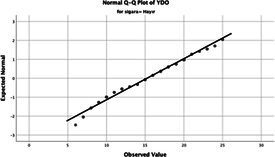	
Health perception	Good	138	48.4	38 (27.5%)	*X* = 6.1* p = 0.009	14.3 ± 5.1 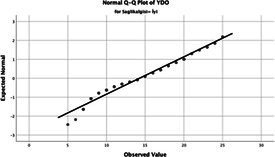	*Z* = −4.9** p < 0.001
	Bad and mid	147	51.6	61 (41.5%)		13.5 ± 4.4 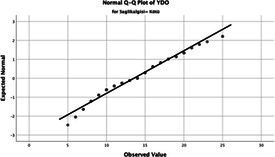	
Income	Below minimum wage	188	66	72 (38.3%)	*X* = 3.1* p = 0.05	13.6 ± 4.8 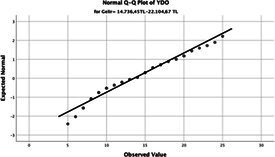	*Z* = −1.8** p = 0.07
	Above minimum wage	97	34	27 (27.8%)		14.5 ± 4.5 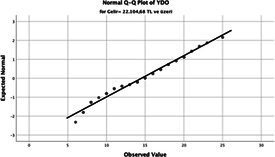	
Places	Malatya	264	92.6	92 (34.8%)	*X* = 0.02* p = 0.5	13.8 ± 4.8 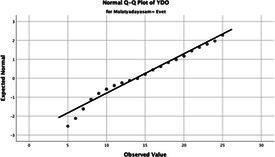	*Z* = −1.4** p = 0.2
	Other	21	7.4	7 (33.3%)		15.3 ± 4.0 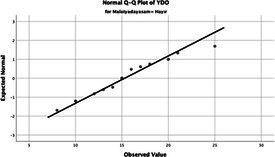	
Correlations between scales					S.rho = −0.24**** p < 0.001		S.rho = −0.24**** p < 0.001

*Chi-square test.

**Mann–Whitney Test.

***Kruskal-Wallis Test.

****Spearman’s correlation.

A multivariable logistic regression analysis was performed to identify factors associated with the presence of voice handicap. Age, educational level, smoking status, presence of chronic disease, use of psychiatric medication, and duration of implant treatment were included in the analysis. The explanatory power of the model was evaluated using the Nagelkerke R^2^ coefficient, and the model was found to explain 28.6% of the variance in the presence of voice handicap (Nagelkerke *R*^2^ = 0.286). Model fit was assessed using the Hosmer–Lemeshow test, which yielded a statistically significant result (χ^2^ = 16.48, *p* = 0.036). According to the analysis results, the use of psychiatric medication was independently and significantly associated with the presence of voice handicap. Individuals using psychiatric medications had approximately a 3.2-fold higher likelihood of experiencing voice handicap compared with those not using such medications (OR = 3.19, 95% CI: 1.63–6.23, *p* = 0.001). Educational level made a statistically significant overall contribution to the model (*p* < 0.001). A significantly increased risk of voice handicap was observed in a specific educational category (OR = 3.20, 95% CI: 1.14–8.95, *p* = 0.027). No statistically significant associations were found between age, smoking status, or presence of chronic disease and the presence of voice handicap (*p* > 0.05). The effect of implant treatment duration on voice handicap was of borderline statistical significance (OR = 1.16, *p* = 0.058) (Table 2).

**Table 2:** Multivariable logistic regression analysis of factors associated with voice handicap.

Variable	*B*	OR (Exp(B))	95% confidence interval	*p*
Age (years)	−0.008	0.99	0.94–1.05	0.770
Education level (overall)	–	–	–	<0.001
Education level (1)	−0.663	0.51	0.19–1.41	0.197
Education level (2)	−0.607	0.55	0.20–1.52	0.247
Education level (3)	0.038	1.04	0.33–3.24	0.948
Education level (4)	1.162	3.20	1.14–8.95	0.027
Education level (5)	−0.679	0.51	0.18–1.44	0.201
Smoking status (yes)	0.309	1.36	0.76–2.45	0.300
Presence of chronic disease	−0.168	0.85	0.45–1.61	0.609
Psychiatric medication use	1.159	3.19	1.63–6.23	0.001
Implant treatment duration (years)	0.152	1.16	0.99–1.36	0.058

*Hosmer–Lemeshow goodness-of-fit test: *p* > 0.05.

A multivariable linear regression analysis was conducted to determine the independent effects of voice handicaps and various sociodemographic and clinical variables on life satisfaction. In the analysis, the life satisfaction score was included as the dependent variable, while the Voice Handicap Index, age, educational level, smoking status, presence of chronic disease, use of psychiatric medication, and duration of implant treatment were entered into the model as independent variables. According to the analysis results, smoking status was identified as an independent and statistically significant predictor of life satisfaction (β = 0.279, *p* < 0.001), indicating that life satisfaction scores differed significantly between individuals who smoked and those who did not. Although a negative association was observed between the Voice Handicap Index and life satisfaction, this relationship did not reach statistical significance (β = −0.105, *p* = 0.091). Similarly, age, educational level, presence of chronic disease, use of psychiatric medication, and duration of implant treatment were not found to have independent and significant effects on life satisfaction (*p* > 0.05). No multicollinearity was detected in the model, with VIF values below 5 for all variables (Table 3).

**Table 3:** Multivariable linear regression analysis of factors associated with life satisfaction.

Variable	*B*	Std. Error	β	*t*	*p*	95% CI for B (Lower–Upper)	VIF
Constant	13.098	4.065	–	3.222	0.001	5.095–21.100	–
Voice Handicap Index (VHI)	−0.064	0.038	−0.105	−1.698	0.091	−0.139 to 0.010	1.194
Age	−0.075	0.050	−0.096	−1.498	0.135	−0.173 to 0.024	1.279
Education level	0.124	0.161	0.045	0.765	0.445	−0.194 to 0.441	1.075
Smoking status	2.648	0.572	0.279	4.629	<0.001	1.522–3.774	1.131
Presence of chronic disease	0.223	0.608	0.022	0.367	0.714	−0.974 to 1.420	1.165
Psychiatric medication use	0.691	0.684	0.060	1.010	0.313	−0.656 to 2.037	1.102
Implant treatment duration (years)	0.170	0.154	0.070	1.103	0.271	−0.133 to 0.474	1.247

Dependent variable: Life Satisfaction Score (LS).

Model fit: Adjusted *R*^2^ = (to be reported from Model Summary).

Multicollinearity: Not detected (all VIF values < 5).

## Discussion

### Communication-related functional outcomes in later life

The present study highlights perceived voice handicap as an important communication-related functional outcome in older adults undergoing dental implant treatment. While oral rehabilitation is traditionally evaluated in terms of mastication and esthetics, the findings suggest that changes in speech-related functions constitute a relevant dimension of functional aging. From a gerontological perspective, communication ability represents a key component of autonomy, social participation, and psychosocial well-being in later life (Rouxel et al., 2016; Cruice et al., 2005). Accordingly, perceived limitations in voice may reflect broader age-related challenges in functional adaptation rather than isolated speech difficulties.

The observed negative association between perceived voice handicap and life satisfaction supports the view that communication-related functions are closely intertwined with subjective well-being in older age. This finding is consistent with previous studies emphasizing that reduced communicative confidence may limit social engagement and contribute to lower life satisfaction among older adults (Rouxel et al., 2016; Cruice et al., 2005). However, the absence of an independent effect of voice handicap on life satisfaction in multivariable analysis suggests that this relationship is complex and likely mediated by contextual and behavioral factors rather than being purely linear.

The attenuation of this association in adjusted models should not be interpreted as diminishing the clinical relevance of voice handicap, but rather as reflecting the interconnected nature of functional, social, and psychological well-being in later life. The modest bivariate correlation (rho = −0.24) may encompass both direct effects of communication difficulties on quality of life and shared variance with unmeasured mediating factors such as social isolation, health literacy, and adaptive capacity. This pattern aligns with gerontological frameworks emphasizing that perceived voice limitations are embedded within a broader constellation of vulnerability indicators including low educational attainment, psychotropic medication use, and reduced social engagement.

### Multidisciplinary determinants of voice handicap

One of the strengths of this study lies in its multidisciplinary interpretation of voice-related outcomes following dental implant treatment. From a dental perspective, implant-supported prostheses alter oral anatomy and articulation dynamics, particularly during the early phases of adaptation (Heydecke et al., 2004; Lundqvist et al., 1992). While these changes may objectively improve oral function, they may simultaneously challenge previously established speech motor patterns, especially in older adults with reduced adaptive reserve.

From the standpoint of speech and language therapy, perceived voice handicap can be understood as a subjective response to altered phonatory and articulatory coordination. Changes in tongue positioning, palatal contact, and oral airflow following implant placement may affect speech clarity and vocal effort, leading individuals to perceive their voice as less reliable in communicative contexts. Importantly, such perceptions may persist even in the absence of clinically evident speech pathology, underscoring the value of patient-reported outcome measures such as the VHI-10 (Jacobson et al., 1997; Rosen et al., 2004; Kılıç et al., 2008).

Gerontologically, these findings align with models of functional vulnerability in later life. Aging is associated with reduced physiological reserve and diminished capacity to compensate for sudden structural or functional changes. In this context, dental implant treatment may act as a functional stressor that reveals latent vulnerabilities in communication-related systems. The relatively high prevalence of perceived voice handicap observed in this study supports this interpretation.

### Psychosocial and contextual vulnerabilities

Educational level and place of residence emerged as significant factors associated with perceived voice handicap. Individuals with lower educational attainment and those living in rural areas reported higher levels of voice-related limitation. These findings may reflect differences in health literacy, access to follow-up care, and opportunities for communicative practice. Nutbeam’s framework on health literacy emphasizes that individuals with limited resources may struggle to interpret bodily changes and adapt to health interventions (Nutbeam, 2008). In the present context, insufficient understanding of the normal adaptation process following implant treatment may amplify negative voice perceptions.

Social interaction also appears to play a buffering role. Participants who reported spending more time with family or friends had lower rates of perceived voice handicap, suggesting that regular communicative engagement may facilitate phonetic adaptation and reinforce communicative confidence. This finding resonates with gerontological theories emphasizing the protective role of social participation in maintaining functional capacity in later life (Rouxel et al., 2016).

### Psychiatric medication and functional vulnerability

The strong association between psychiatric medication use and perceived voice handicap represents one of the most clinically relevant findings of this study. Older adults using psychiatric medications were significantly more likely to report voice-related limitations. From a speech and language therapy perspective, medication-related side effects such as xerostomia, reduced oral motor control, and altered sensory feedback may impair phonation and articulation. Dental literature similarly highlights the impact of systemic medications on oral and vocal function (Baumgartner et al., 2015).

Gerontologically, psychiatric medication use may serve as an indicator of increased functional vulnerability. Older adults with mental health conditions often experience cumulative physiological and psychosocial burdens that reduce adaptive capacity. In this context, voice handicap may represent a visible manifestation of broader functional fragility rather than an isolated outcome. These findings underscore the importance of interdisciplinary assessment and follow-up in older implant recipients, particularly those with complex medication profiles.

### Adaptation, expectations, and prosthetic history

The higher prevalence of perceived voice handicap among individuals with prior removable denture use highlights the role of expectations and adaptation processes. Patients with long-standing denture experience may have developed stable speech patterns and formed idealized expectations regarding implant-supported prostheses. When post-treatment speech does not align with these expectations, perceived dissatisfaction may arise, even in the presence of functional improvement.

This interpretation is consistent with earlier studies demonstrating that patient expectations strongly influence perceived treatment outcomes (Allen et al., 1999; Yao et al., 2014; Berretin-Felix et al., 2008). From a speech therapy and gerontological perspective, adaptation to new oral structures may require time, guided practice, and reassurance, particularly in older adults with reduced neuroplasticity. The finding that life satisfaction increased with longer implant treatment duration supports the notion that adaptation is a gradual process that extends beyond the immediate post-treatment period (Heydecke et al., 2004).

### Clinical and interdisciplinary implications

Taken together, the findings of this study emphasize the need for a multidisciplinary approach to dental implant treatment in older adults. Collaboration between dental professionals, speech and language therapists, and gerontologists may enhance the identification and management of communication-related challenges following oral rehabilitation. Routine screening for perceived voice handicap, patient education regarding the adaptation process, and targeted interventions for vulnerable subgroups may contribute to improved long-term outcomes.

Beyond its immediate clinical relevance, perceived voice-related functional limitation may reflect broader processes of functional adaptation in later life. From an aging perspective, voice-related difficulties can influence everyday communication, social engagement, and self-confidence, thereby shaping overall psychosocial well-being. In this sense, voice handicap should not be viewed solely as an isolated communication problem but rather as a functional indicator of increased vulnerability, particularly among older adults exposed to additional educational or pharmacological challenges. The present findings suggest that communication-focused functional outcomes following oral rehabilitation deserve greater attention within aging research, as they may contribute meaningfully to understanding adaptive capacity and quality of life in later life.

### Limitations and future directions

Several limitations of this study should be acknowledged. First, the cross-sectional design does not allow for causal inferences regarding the relationship between perceived voice-related functional limitations and life satisfaction. It is plausible that lower life satisfaction predisposes individuals to greater perceived voice problems, that voice difficulties reduce life satisfaction, or that both are influenced by common underlying factors such as general health status or psychosocial vulnerability. Longitudinal studies tracking patients from pre-treatment baseline through post-implant adaptation would better elucidate temporal relationships and identify critical periods for intervention. Second, the use of self-reported measures may be subject to reporting bias; however, validated instruments were employed to capture participants’ subjective experiences, which are central to the constructs under investigation. In addition, the study sample was limited to older adults who had undergone dental implant treatment, which may restrict the generalizability of the findings to other oral rehabilitation contexts. Despite these limitations, the study provides functionally relevant insights into communication-related outcomes in later life and highlights an underexplored dimension of adaptive capacity in aging populations.

Future research should employ longitudinal designs to track voice handicap and life satisfaction trajectories from pre-treatment through post-implant adaptation. Studies should include objective measures of speech intelligibility and voice quality alongside patient-reported outcomes to better understand the relationship between subjective perception and objective function. Mixed-methods approaches incorporating qualitative interviews would provide deeper insight into how older adults experience and adapt to voice changes following implant treatment. Intervention studies testing speech therapy protocols or patient education programs could establish evidence-based approaches to supporting communicative adaptation. Finally, studies should examine whether voice handicap screening and targeted intervention improve long-term patient satisfaction and quality of life outcomes.

## Conclusion

This study shows that perceived voice-related limitations are strongly linked to life satisfaction in older adults who have received dental implant treatment. The findings reveal that a higher level of voice handicap corresponds with lower life satisfaction, even when accounting for sociodemographic and clinical factors. This suggests that after dental implant treatment, both oral functions and personal voice-related experiences can significantly affect the overall well-being of older people.

The results stress that voice-related issues should be taken seriously in the assessment and follow-up of dental implant treatment in older adults. In clinical practice, identifying voice handicaps early and making appropriate referrals may help improve life satisfaction. Future studies should use longitudinal designs and larger samples to better explore the relationships between dental implant treatment, voice handicaps, and life satisfaction. It is also recommended to include qualitative approaches for deeper insights into patients’ personal experiences.

## Data Availability

The original contributions presented in the study are included in the article/supplementary material, further inquiries can be directed to the corresponding author.
